# Case Report: Rare Acute Abdomen: Focal Nodular Hyperplasia With Spontaneous Rupture

**DOI:** 10.3389/fonc.2022.873338

**Published:** 2022-07-13

**Authors:** Ying Si, Bo Sun, Ting Zhao, Ke Xiao, Dong-Xia Zhao, Yong-Mao Huang

**Affiliations:** ^1^ Department of Infectious Diseases, The Affiliated Hospital of Southwest Medical University, Luzhou, China; ^2^ Department of Ultrasound, The Affiliated Hospital of Southwest Medical University, Luzhou, China

**Keywords:** focal nodular hyperplasia (FNH), liver, rupture, acute abdomen, surgery

## Abstract

Focal nodular hyperplasia (FNH) of the liver is a benign lesion characterized by hypertrophic nodules with central star-shaped fibrous scars. The etiology and pathogenesis of FNH are not completely understood. A 43-year-old man was hospitalized because of acute abdominal pain. Emergency computed tomography(CT) showed hepatic tumor rupture and bleeding. The patient’s condition improved following arteriographic embolization to stop bleeding. Laparotomy confirmed spontaneous rupture and hemorrhage of focal hyperplasia and the patient remains asymptomatic after an uneventful recovery. FNH with spontaneous rupture and bleeding is extremely rare. Currently, there is no unified management standard for FNH and most previous studies recommend observation and follow-up. We recommend consideration of surgical treatment of cases with spontaneous rupture and bleeding.

## Introduction

Focal nodular hyperplasia (FNH) is the second most common benign liver tumor and accounts for approximately 8% of all primary hepatic tumors. It is thought to be a hyperplastic response to increased blood flow in an arterial malformation rather than a true neoplasm (1, 2). Spontaneous hemorrhage of an FNH is very rare, and only 10 cases have been reported (3). This patient with spontaneous rupture and bleeding of a FNH in the right liver was successfully treated by second step surgical treatment.

## Case presentation

**Figure 1:**
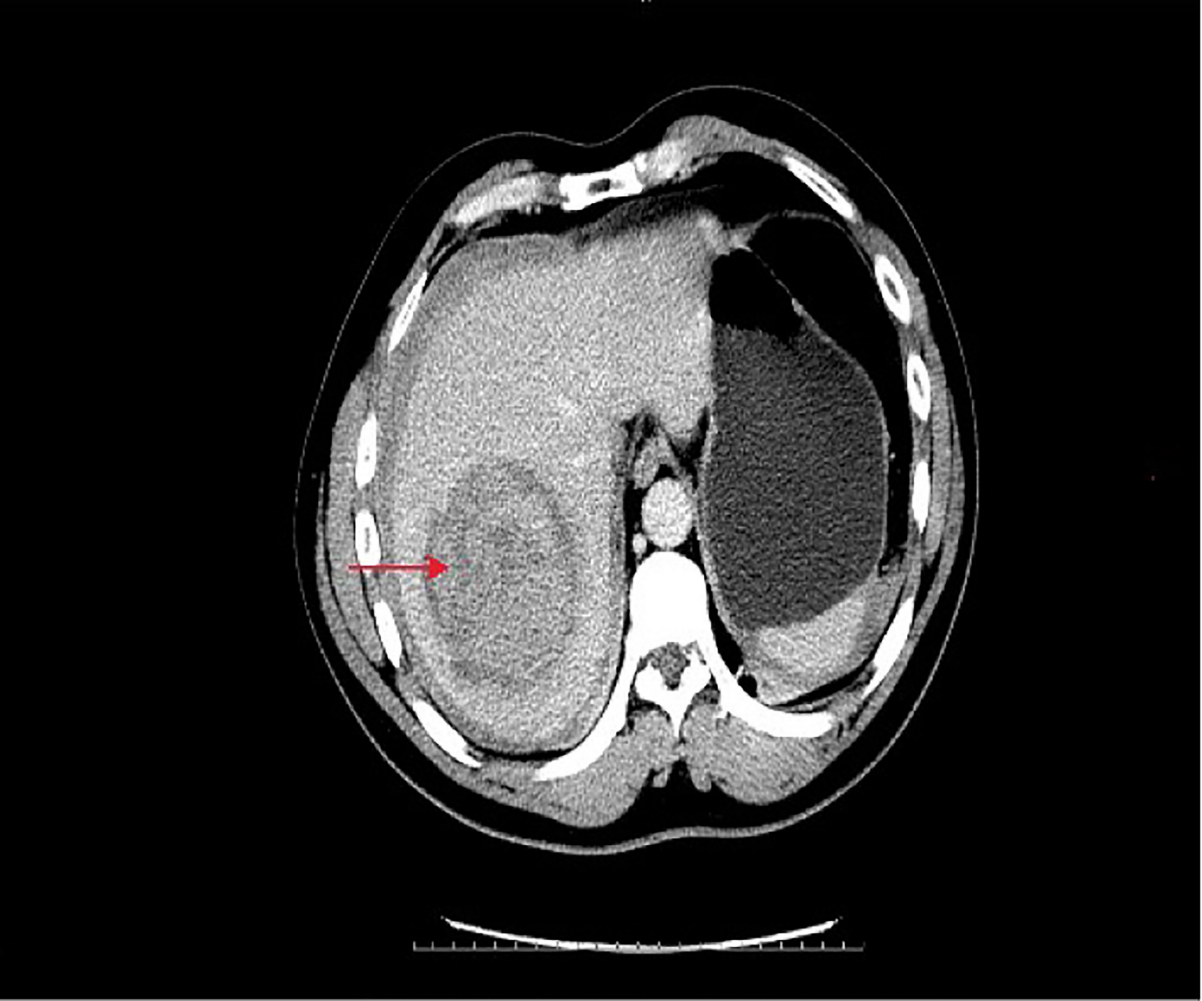
Emergency CT of the abdomen showed a mixed density mass of about 8*8 cm in the right lobe of the liver. The lesion was near the right hepatic artery and free liquid density was visible around it.

**Figure 2:**
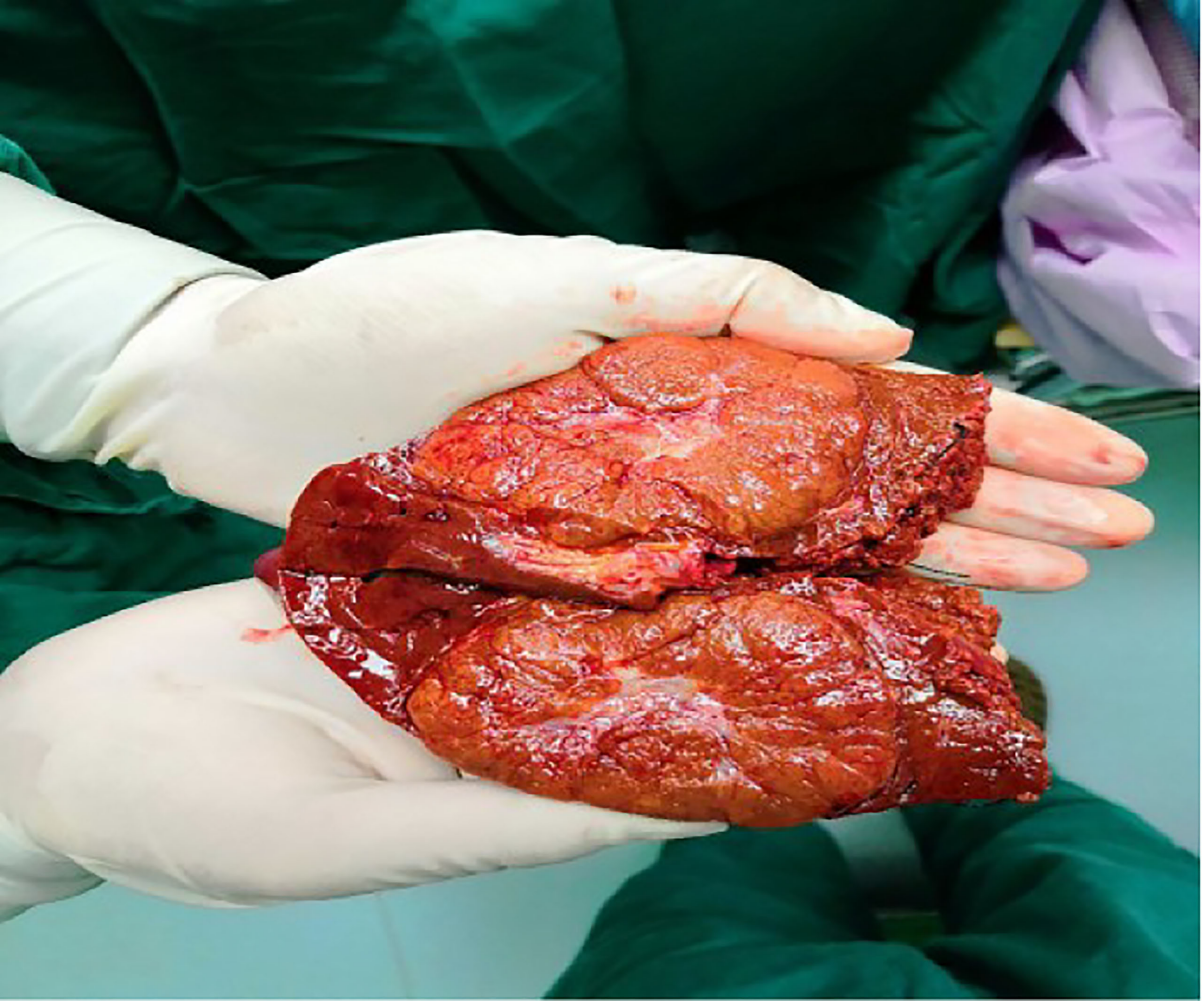
The tumor is nodular to the naked eye, and the cut surface is grayish yellow, and blood is visible.

**Figure 3:**
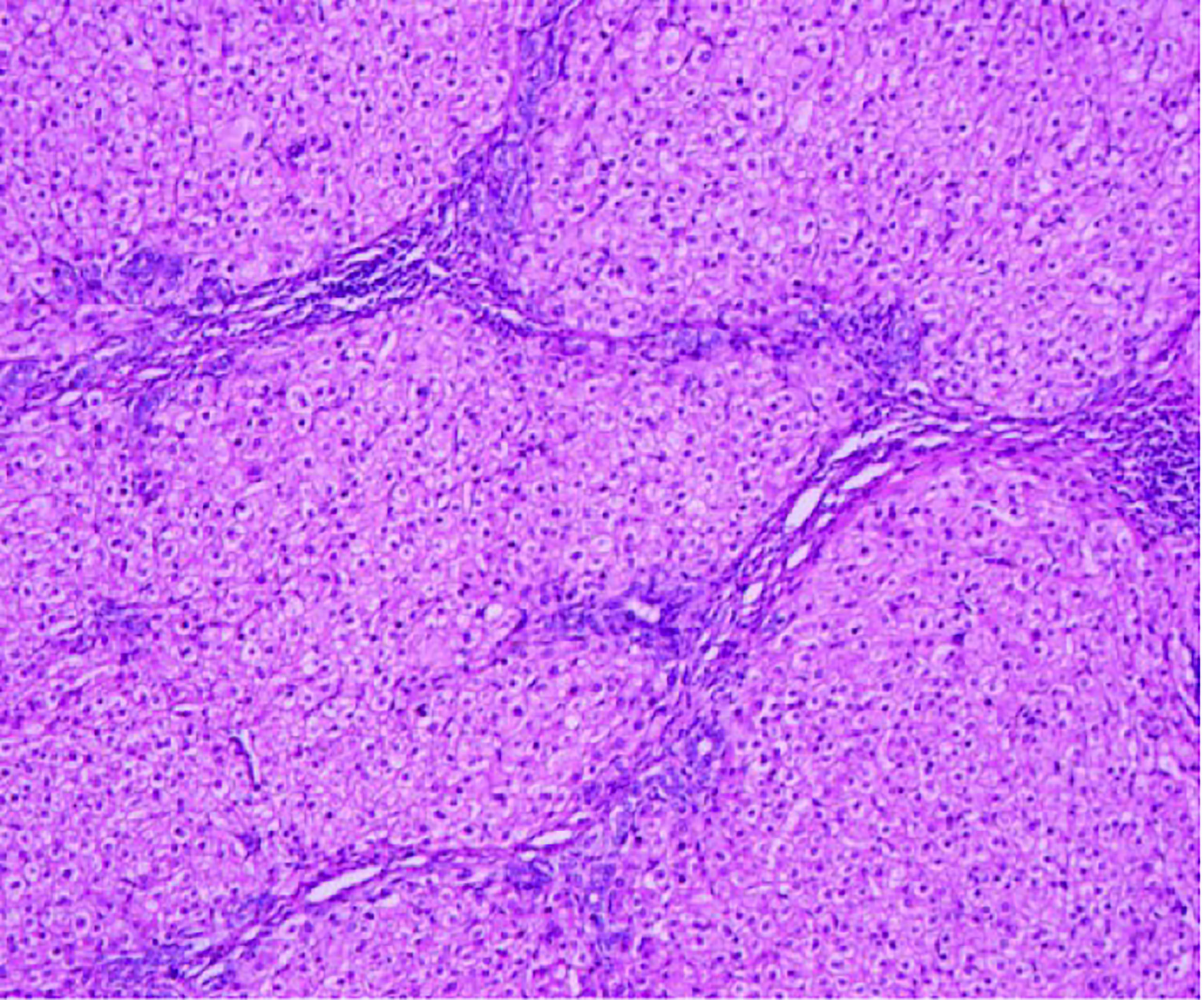
Hematoxylin-eosin staining showed hepatocyte proliferation and vasodilation, and no atypical cells.

**Figure 4:**
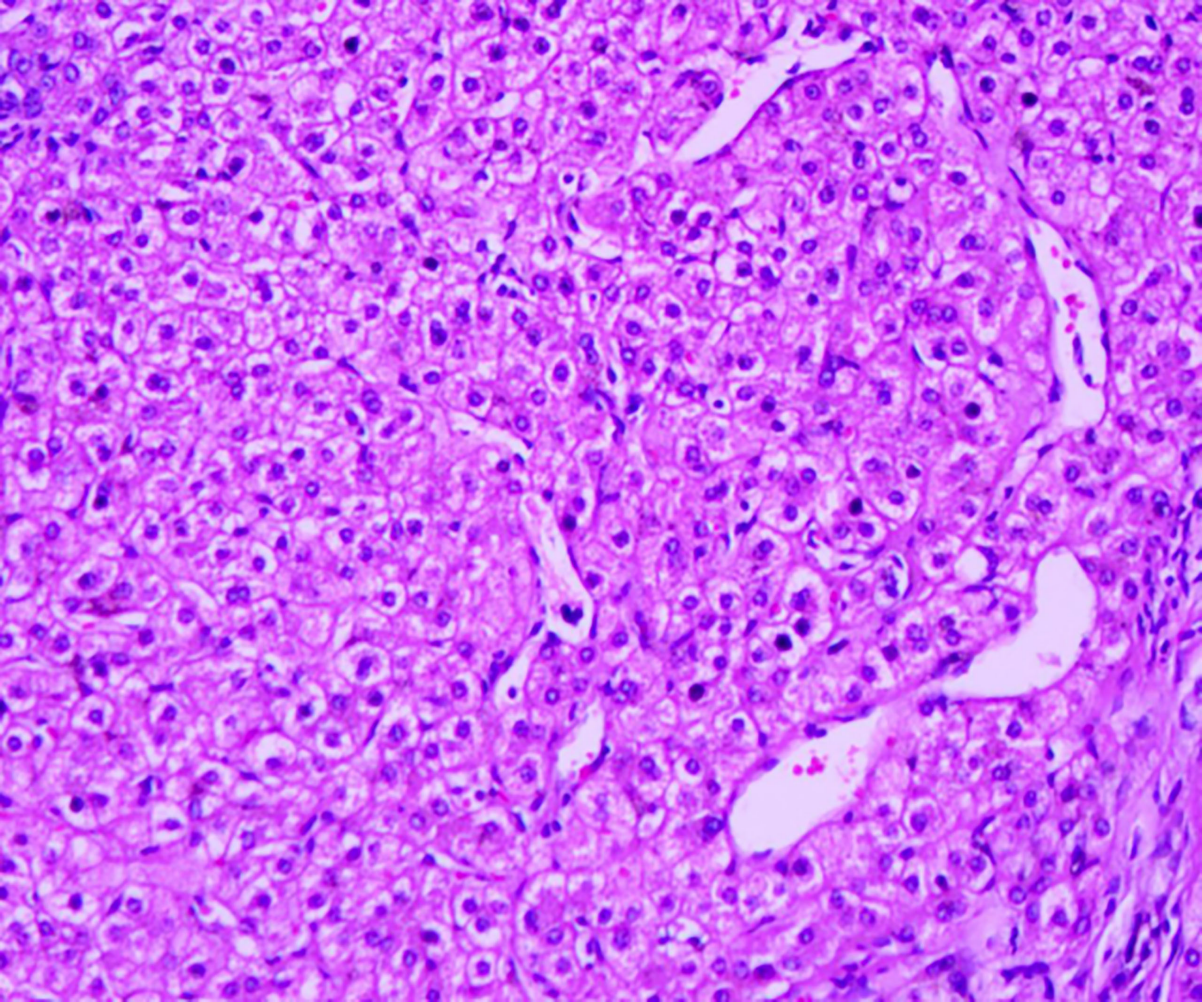
Hematoxylin-eosin staining showed hepatocyte proliferation and vasodilation, and no atypical cells.

**Figure 5:**
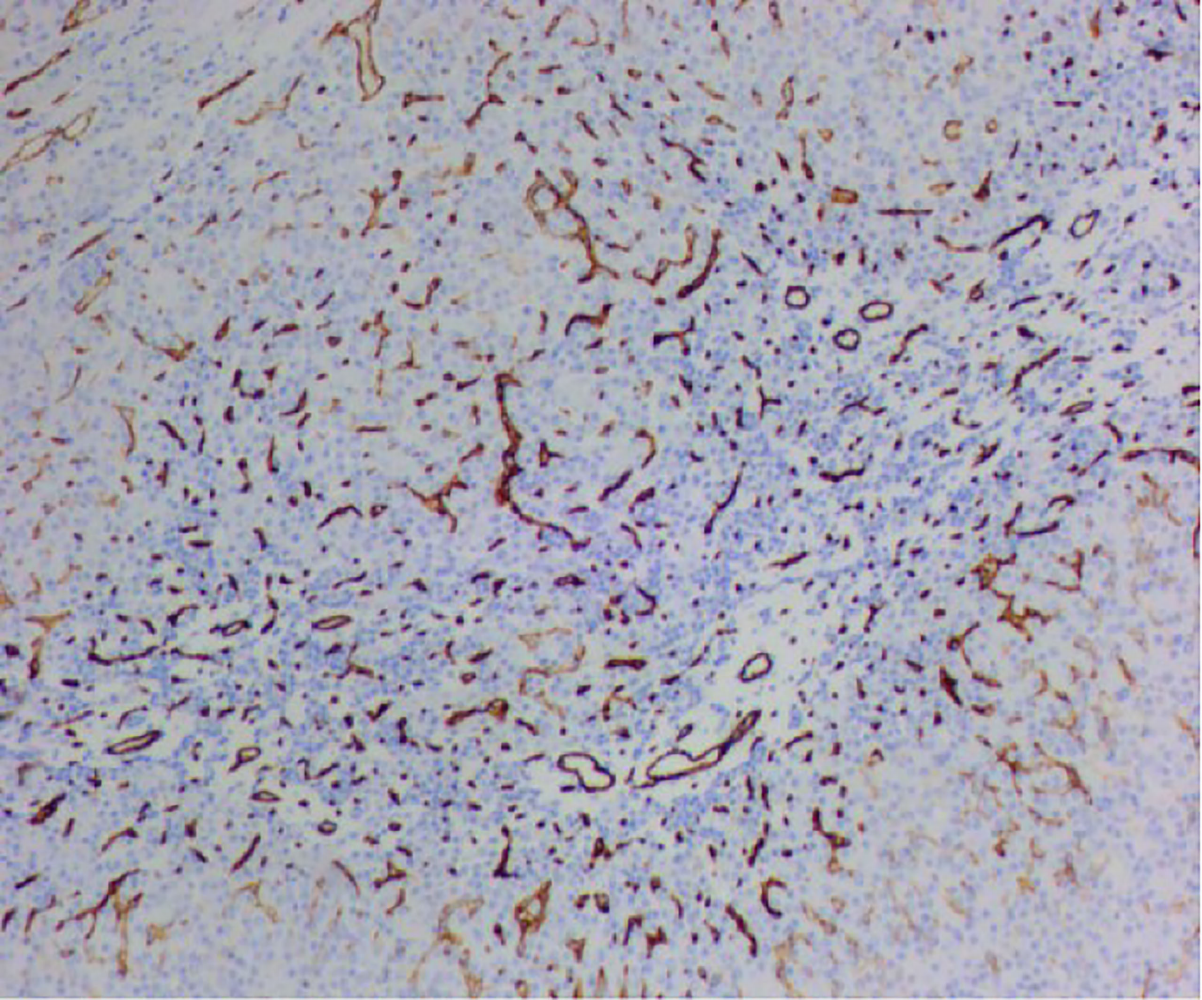
Immunohistochemistry showed CK19 (focal +).

**Figure 6:**
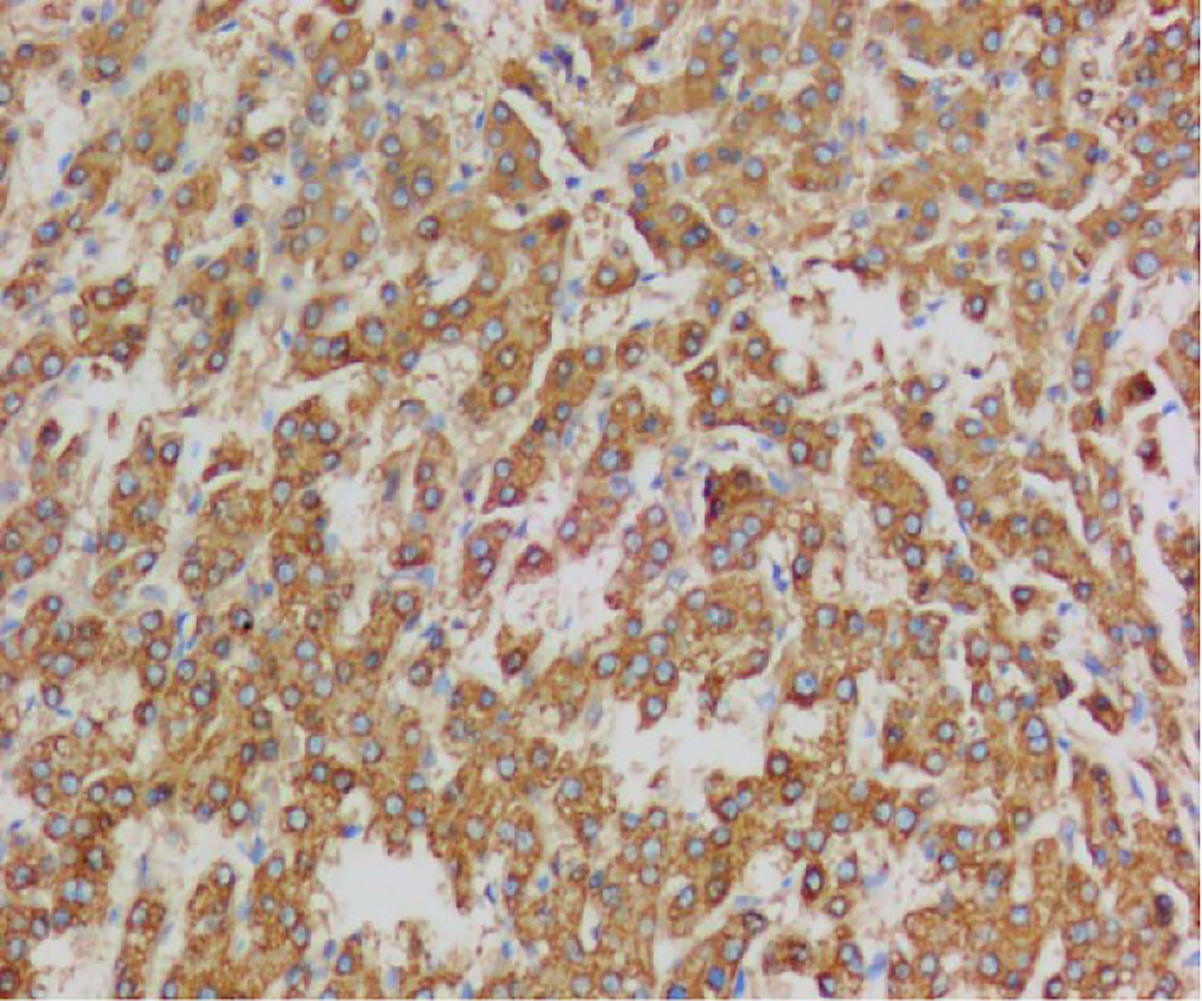
Immunohistochemistry showed CD34(+).

A 43-year-old Chinese man was admitted to the hospital because of acute abdominal pain. He had no history of trauma, was in good health, and had been an intermittent alcohol user for about 20 years. Physical examination revealed no palpable mass or tenderness in the right upper abdomen. Blood routine showed that Hemoglobin was 142g/L.Alanine aminotransferase(502.6/U/L)and aspartate aminotransferase(527.9/U/L)were significantly increased. Blood coagulation tests and tumor-marker levels were normal. Abdominal enhanced computed tomography (CT) showed a round, approximately8.0cm×8.0cm mass with a slightly blurred border in the right lobe of the liver, and blood around the liver. A tumor hemorrhage was suspected (**Figure 1**) and as continued bleeding could not be ruled out, the patient underwent hepatic arteriographic embolization in the emergency department. Intraoperative angiographic findings the tumor was stained in irregular mass, with irregular outer border and widened perihepatic shadow. During the operation, lipiodol and gelatin sponge particles were used to embolize the responsible blood supply artery of the tumor. Re-imaging after embolization showed that the imaging of tumor supplying arteries was significantly reduced, the tumor staining range was significantly reduced, and the embolic agent was well deposited and the patient’s condition improved after 2 weeks of conservative treatment. After preoperative and intraoperative evaluation, the patient underwent right hemihepatectomy. Intraoperative exploration revealed that most of the tumor was located in segment VII and VIII, and a small part was located in segment V, adjacent to the right hepatic artery. Tumor size is about 8.0cm×8.0cm cm with an incomplete capsule, the boundary was clear, and an old blood accumulation was seen around the liver. The resected tumor was round, with clear boundaries and contained a hematoma. Pathologic examination of hematoxylin–eosin-stained tissue showed hepatocyte proliferation and vasodilation, and no atypical hyperplasia (**Figures 2**). Immunohistochemistry showed focally positive CK19 and CD34 cells consistent with capillary formation (**Figures 3**). The pathological features resulted in a final diagnosis of FNH with spontaneous rupture and bleeding. The patient recovered uneventfully and remains asymptomatic for 2 years. **Figure 4**, **5**, **6**

## Discussion

FNH is the second most prevalent benign liver tumor after hepatic cavernous hemangioma. The incidence is highest in those 20–50 years of age, but FNH can occur at any age (4, 5). It most often occurs in women of childbearing age with a history of oral contraceptives (6, 7). The etiology is not fully understood, but vascularization by an anomalous artery, reactive hyperplasia after hepatocellular injury induced by vasculitis, or aberrant, increased blood flow have been implicated (8–10).

Previous cases of FNH with intraperitoneal hemorrhage, including this patient, reported in English-language publications are shown in **Table 1** (3, 14–20). This review reviewed 11 patients, including 2 males and 9 females. The incidence was mainly female, with an average age of 31.7 years. As most FNH patients have no symptoms, most lesions are found by accident. Most symptomatic patients present with abdominal pain, discomfort in the right upper abdomen, and nausea. Very few have palpable masses (21–26). Liver enzyme values are abnormal in the serum of 10% to 14% of patients (27).Abdominal ultrasound, CT, and magnetic resonance imaging (MRI) with radioactive labels may reveal star scars (28).MRI has higher sensitivity and specificity for diagnosis of FNH than CT and abdominal ultrasound, especially magnetic resonance cholangiopancreatography (29, 30). Contrast-enhanced ultrasound with sonazoid may provide hemodynamic information of the vascular pattern and Kupffer-phase imaging improves diagnostic confidence and is effective for follow-up in clinical practice (31).

**Table 1:** Documented patients of hemorrhage caused by FNH.

First author (year)	Age (years)/sex	Diameter (mm)	Location	No	Imaging findings	Treatment	Outcome	(Refs.)
Mays ET(1974)	26/F	100	Right lobe	1	NR	Surgery	NR	(11)
Becker YT(1995)	18/F	45	Right lobe	2	NR	Surgery	NR	(12)
Hardwigsen J(2001)	37/F	50	Right lobe	1	NR	Surgery	NR	(13)
Bathe OF(2003)	27/F	60	Right lobe	1	HHAF	Surgery	Alive/18 mo	(14)
Rahili A(2005)	35/F	98	Lobus caudatus	1	HHAF	Surgery	Alive/78 mo	(15)
Chang SK(2005)	42/F	100	Right lobe	1	HHAF	Surgery	NR	(16)
Demarco MP(2006)	37/F	52	Left lobe	4	HHAF	Surgery	NR	(17)
Li T (2006)	26/F	150	Left robe	NR	HHAF	Surgery	Alive/8 mo	(18)
Yajima D(2013)	23/F	10	Right lobe	1	NR	Revealed at autopsy	Dead	(19)
Kinoshita M (2016)	35/M	80	Right lobe	1	HHAF	Surgery	Alive/48 mo	(3)
Present study (2020)	43/M	80	Right lobe	1	HHAF	Surgery	Alive/to date	(-)

M, male; F, female; mo, months; No, number; HHAF, high-density hematoma area formed; NR, not reported.

The occurrence of spontaneous rupture and bleeding of FNH might be explained as follows: Bleeding in FNH patients may be the result of vascular malformations and intratumoral pressure associated (2, 9, 10). Increased intratumoral pressure compresses the malformed blood vessels, eventually leading to results in spontaneous bleeding. For this patient, he had a history of alcoholic hepatitis, and the liver tissue was fragile. The tumor was located around the liver and adjacent to the right hepatic artery and was large and vulnerable to external force. Therefore, the combined effect of the above factors may have caused the rupture of the tumor.

Nguye et al. (32) described FNH as having two pathologies classical and non-classical. Non-classical FNH includes telangiectasia, mixed hyperplasia, and adenoma-like characteristics. Classical FNH accounts for the vast majority of diagnoses. Fabre et al. (33) proposed FNH histology scoring criteria including four main characteristics (fibrous elements, thick-walled blood vessels, hyperplastic small bile ducts, and nodules) and two minor characteristics (dilatation of liver blood sinuses and sinus fibrosis). FNH can be diagnosed if three of the four main characteristics, two main characteristics, and one or two minor characteristics are present. The presence of two or fewer major characteristics does not support a diagnosis of FNH. FNH currently has no specific immunohistochemical markers. CK19 and CK56 are markers of liver precursor cells and bile duct epithelium and CK7 is a marker of immature hepatocytes. The combined use of CK7 and CK19 is helpful for the diagnosis of FNH. CD34 is a marker of vascular endothelium, and because dilated arteries with thick walls or cavernous hemangioma occur in FNH lesions, CD34 staining is often positive. CD19 is a membrane antigen associated with cell proliferation. FNH is a local vascular malformation of the liver with increased perfusion that results in abnormal proliferation of local hepatocytes and formation of nodular lesions. Therefore, CD19 staining is often positive. There are reports in the literature that β-catenin can activate glutamine synthetase (GS), which results in typical map-like staining. Therefore, GS staining may assist in the diagnosis of FNH (34, 35).

At present, there is no consensus on the standard treatment of FNH, which is a benign lesion with no underlying malignancy. Most recommendations are for follow-up of asymptomatic patients (36). Surgery is the mainstay of treatment for patients with symptoms, enlarged lesions, and imaging indeterminate lesions during follow-up. Rupture and bleeding of liver tumors is a life-threatening condition. Emergency arteriographic embolization of unexplained hepatic mass hemorrhage can successfully control bleeding in 99% of patients (13). After the patient’s condition improves, the second-stage mass can be removed and the condition diagnosed. In our experience, combined first-stage interventional embolization and second-stage mass resection can be used as the standard treatment for FNH rupture and bleeding.

## Conclusion

Spontaneous rupture and bleeding of FNH is very rare, but should be fully considered in patients who experience sudden abdominal pain during follow-up.

## Data Availability Statement

The original contributions presented in the study are included in the article/Supplementary Material. Further inquiries can be directed to the corresponding author.

## Ethics Statement

Written informed consent was obtained from the participant for the publication of this case report.

## Author Contributions

YS wrote all drafts. BS discussed the meaning of the draft. TZ and KX collect all the references. D-XZ carried out the pathology and collected the clinical data. Y-MH offered conception and finalized the draft. All authors read and approved the final manuscript.

## Conflict of Interest

The authors declare that the research was conducted in the absence of any commercial or financial relationships that could be construed as a potential conflict of interest.

## Publisher’s Note

All claims expressed in this article are solely those of the authors and do not necessarily represent those of their affiliated organizations, or those of the publisher, the editors and the reviewers. Any product that may be evaluated in this article, or claim that may be made by its manufacturer, is not guaranteed or endorsed by the publisher.

## Abbreviations

FNH, Focal nodular hyperplasia; CT, Computed tomography; MRI, magnetic resonance imaging; CEUS, contrast-enhanced ultrasound; GS, glutamine synthetase; RFA, radiofrequency ablation; TAE, transarterial embolization; PVA, polyvinyl alcohol.

## References

[1] Vilgrain V. Focal Nodular Hyperplasia. Eur J Radiol (2006) 58(2):236–45. doi: 10.1016/j.ejrad.2005.11.043 16414229

[2] Oldhafer KJ Habbel V Horling K Makridis G Wagner KC. Benign Liver Tumors. Visceral Med (2020) 36(4):292–303. doi: 10.1159/000509145 PMC7506257 33005655

[3] Kinoshita M Takemura S Tanaka S Hamano Ito G Aota T. Ruptured Focal Nodular Hyperplasia Observed During Follow-Up: A Case Report. Surg Case Rep (2017) 3(1):44. doi: 10.1186/s40792-017-0320-4 28315131 PMC5357241

[4] Pekli D Kokas B Bárdos D Fülöp A Pajor P Hahn O. A Focalis Nodularis Hyperplasia Multimodális Kezelése. Orvosi Hetilap (2022) 163(15):606–12. doi: 10.1556/650.2022.32439 35398818

[5] Assy N Nasser G Djibre A Beniashvili Z Elias S Zidan J. Characteristics of Common Solid Liver Lesions and Recommendations for Diagnostic Workup. World J Gastroenterol (2009) 15(26):3217–27. doi: 10.3748/wjg.15.3217 PMC2710776 19598296

[6] Herman P Pugliese V Machado MA Montagnini AL Salem MZ Bacchella T. Hepatic Adenoma and Focal Nodular Hyperplasia: Differential Diagnosis and Treatment. World J Surg (2000) 24(3):372–6. doi: 10.1007/s002689910059 10658075

[7] Pain JA Gimson AE Williams R Howard ER. Focal Nodular Hyperplasia of the Liver: Results of Treatment and Options in Management. Gut (1991) 32(5):524–7. doi: 10.1136/gut.32.5.524 PMC1378930 2040476

[8] Chiorean L Cui XW Tannapfel A Franke D Stenzel M Kosiak W. Benign Liver Tumors in Pediatric Patients - Review With Emphasis on Imaging Features. World J Gastroenterol (2015) 21(28):8541–61. doi: 10.3748/wjg.v21.i28.8541 PMC4515836 26229397

[9] Franchi-Abella S Branchereau S. Benign Hepatocellular Tumors in Children: Focal Nodular Hyperplasia and Hepatocellular Adenoma. Int J Hepatol (2013) 2013:215064. doi: 10.1155/2013/215064 23555058 PMC3608344

[10] European Association for the Study of the Liver (EASL). EASL. Clinical Practice Guidelines on the Management of Benign Liver Tumours. J Hepatol (2016) 65(2):386–98. doi: 10.1016/j.jhep.2016.04.001 27085809

[11] Hsueh KC Fan HL Chen TW Chan DC Yu JC Tsou SS. Management of Spontaneously Ruptured Hepatocellular Carcinoma and Hemoperitoneum Manifested as Acute Abdomen in the Emergency Room. World J Surg (2012) 36(11):2670–6. doi: 10.1007/s00268-012-1734-6 22864567

[12] Mays ET Christopherson WM Barrows GH. Focal Nodular Hyperplasia of the Liver. Possible Relationship to Oral Contraceptives. Am J Clin Pathol (1974) 61(6):735–46. doi: 10.1093/ajcp/61.6.735. 4364925

[13] Becker YT Raiford DS Webb L Wright JK Chapman WC Pinson CW. Rupture and Hemorrhage of Hepatic Focal Nodular Hyperplasia. Am Surg (1995) 61(3):210–4. doi: 00000478-199503000-00018. 7887531

[14] Hardwigsen J Pons J Veit V Garcia S Le Treut YP. A Life-Threatening Complication of Focal Nodular Hyperplasia. J Hepatol (2001) 35(2):310–2. doi: 10.1016/S0168-8278(01)00096-4 11580160

[15] Bathe OF Mies C Franceschi D Casillas J Livingstone AS. Massive Hemorrhage and Infarction Complicating Focal Nodular Hyperplasia of the Liver. HPB (Oxford) (2003) 5(2):123–6. doi: 10.1080/13651820310000 PMC2020571 18332970

[16] Rahili A Cai J Trastour C Juwid A Benchimol D Zheng M. Spontaneous Rupture and Hemorrhage of Hepatic Focal Nodular Hyperplasia in Lobus Caudatus. J Hepatobiliary Pancreat Surg (2005) 12(2):138–42. doi: 10.1007/s00534-004-0936-1 15868078

[17] Chang SK Chung YF Thng CH Loo HW. Focal Nodular Hyperplasia Presenting as Acute Abdomen. Singapore Med J (2005) 46(2):90–2. 15678292

[18] Demarco MP Shen P Bradley RF Levine EA. Intraperitoneal Hemorrhage in a Patient With Hepatic Focal Nodular Hyperplasia. Am Surg (2006) 72(6):555–9. doi: 10.1177/000313480607200620 16808214

[19] Li T Qin LX Ji Y Sun HC Ye QH Wang L. Atypical Hepatic Focal Nodular Hyperplasia Presenting as Acute Abdomen and Misdiagnosed as Hepatocellular Carcinoma. Hepatol Res (2007) 37(12):1100–5. doi: 10.1111/j.1872-034X.2007.00164.x 17608671

[20] Yajima D Kondo F Nakatani Y Saitoh H Hayakawa M Sato Y. A Fatal Case of Subcapsular Liver Hemorrhage in Late Pregnancy: A Review of Hemorrhages Caused by Hepatocellular Hyperplastic Nodules. J Forensic Sci (2013) 58 Suppl 1:S253–7. doi: 10.1111/j.1556-4029.2012.02246.x 22900716

[21] Guttmacher AE Marchuk DA White RI Jr. Hereditary Hemorrhagic Telangiectasia. N Engl J Med (1995) 333(14):918–24. doi: 10.1056/NEJM199510053331407 7666879

[22] Buscarini E Danesino C Plauchu H de Fazio C Olivieri C Brambilla G. High Prevalence of Hepatic Focal Nodular Hyperplasia in Subjects With Hereditary Hemorrhagic Telangiectasia. Ultrasound Med Biol (2004) 30(9):1089–97. doi: 10.1016/j.ultrasmedbio.2004.08.004 15550313

[23] Scardapane A Ficco M Sabbà C Lorusso F Moschetta M Maggialetti N. Hepatic Nodular Regenerative Lesions in Patients With Hereditary Haemorrhagic Telangiectasia: Computed Tomography and Magnetic Resonance Findings. Radiol Med (2013) 118(1):1–13. doi: 10.1007/s11547-012-0789-3 22327916

[24] Bonney GK Gomez D Al-Mukhtar A Toogood GJ Lodge JP Prasad R. Indication for Treatment and Long-Term Outcome of Focal Nodular Hyperplasia. HPB (Oxford) (2007) 9(5):368–72. doi: 10.1080/13651820701504173 PMC2225515 18345321

[25] Hsee LC McCall JL Koea JB. Focal Nodular Hyperplasia: What are the Indications for Resection? HPB (Oxford) (2005) 7(4):298–302. doi: 10.1080/13651820500273624 18333211 PMC2043107

[26] Shawcross DL Naoumov N Pachiadakis I Mamais C Williams R Jalan R. Should a Biopsy Precede Liver Resection or Transplantation for Presumed Hepatocellular Carcinoma When the Alfa Fetoprotein is Normal? Transplantation (2004) 77(4):637–8. doi: 10.1097/01.TP.0000109783.98513.E3 15084957

[27] Fioole B Kokke M van Hillegersberg R Rinkes IH. Adequate Symptom Relief Justifies Hepatic Resection for Benign Disease. BMC Surg (2005) 5:7. doi: 10.1186/1471-2482-5-7 15804352 PMC1087495

[28] Grazioli L Morana G Federle MP Brancatelli G Testoni M Kirchin MA. Focal Nodular Hyperplasia: Morphologic and Functional Information From MR Imaging With Gadobenate Dimeglumine. Radiology (2001) 221(3):731–9. doi: 10.1148/radiol.2213010139 11719669

[29] Bieze M van den Esschert JW Nio CY Verheij J Reitsma JB Terpstra V. Diagnostic Accuracy of MRI in Differentiating Hepatocellular Adenoma From Focal Nodular Hyperplasia: Prospective Study of the Additional Value of Gadoxetate Disodium. AJR Am J Roentgenol (2012) 199(1):26–34. doi: 10.2214/AJR.11.7750 22733890

[30] Auer TA Walter-Rittel T Geisel D Schning W Fehrenbach U. HBP-Enhancing Hepatocellular Adenomas and How to Discriminate Them From FNH in Gd-EOB MRI. BMC Med Imaging (2021) 21(1):1–11. doi: 10.1186/s12880-021-00552-0 33588783 PMC7885421

[31] Lee J Jeong WK Lim HK Kim AY. Focal Nodular Hyperplasia of the Liver: Contrast-Enhanced Ultrasonographic Features With Sonazoid. J Ultrasound Med (2018) 37(6):1473–80. doi: 10.1002/jum.14490 29159819

[32] Nguyen BN Fléjou JF Terris B Belghiti J Degott C. Focal Nodular Hyperplasia of the Liver: A Comprehensive Pathologic Study of 305 Lesions and Recognition of New Histologic Forms. Am J Surg Pathol (1999) 23(12):1441–54. doi: 10.1097/00000478-199912000-00001 10584697

[33] Fabre A Audet P Vilgrain V Nguyen BN Valla D Belghiti J. Histologic Scoring of Liver Biopsy in Focal Nodular Hyperplasia With Atypical Presentation. Hepatology (2002) 35(2):414–20. doi: 10.1053/jhep.2002.31103 11826417

[34] Rowan DJ Allende DS Bellizzi AM Gill RM Liu X McKenzie CA. Diagnostic Challenges of Focal Nodular Hyperplasia: Interobserver Variability, Accuracy, and the Utility of Glutamine Synthetase Immunohistochemistry. Histopathology (2021) 79(5):791–800. doi: 10.1111/his.14424 34080211

[35] Bioulac-Sage P Laumonier H Rullier A Cubel G Laurent C Zucman-Rossi J. Over-Expression of Glutamine Synthetase in Focal Nodular Hyperplasia: A Novel Easy Diagnostic Tool in Surgical Pathology. Liver Int (2009) 29(3):459–65. doi: 10.1111/j.1478-3231.2008.01849.x 18803590

[36] Perrakis A Demir R Müller V Mulsow J Aydin Ü Alibek S. Management of the Focal Nodular Hyperplasia of the Liver: Evaluation of the Surgical Treatment Comparing With Observation Only. Am J Surg (2012) 204(5):689–96. doi: 10.1016/j.amjsurg.2012.02.006 22578408

